# MiR-103-3p promotes hepatic steatosis to aggravate nonalcoholic fatty liver disease by targeting of ACOX1

**DOI:** 10.1007/s11033-022-07515-w

**Published:** 2022-05-23

**Authors:** Jiexia Ding, Caixia Xia, Panpan Cen, Siying Li, Lifei Yu, Jing Zhu, Jie Jin

**Affiliations:** Department of Infectious Diseases, Affiliated Hangzhou First People’s Hospital, Zhejiang University School of Medicine, No. 261 Huansha Road, Hangzhou, 310003 Zhejiang Province China

**Keywords:** Nonalcoholic fatty liver disease, miR-103-3p, ACOX1, Reactive oxygen species

## Abstract

**Background:**

Nonalcoholic fatty liver disease (NAFLD) is a major risk factor for hepatocellular carcinoma, and alterations in miRNA expression are related to the development of NAFLD. However, the role of miRNAs in regulating the development of NAFLD is still poorly understood.

**Methods:**

We used qRT-PCR to detect the level of miR-103-3p in both cell and mouse models of NAFLD. Biochemical assays, DCF-DA assays, Oil red O staining and HE staining were used to detect the role of miR-103-3p in NAFLD development. Target genes of miR-103-3p were predicted using the TargetScan database and verified by qRT-PCR, western blot and dual-luciferase assays.

**Results:**

The expression of miR-103-3p increased in both NAFLD model cells and liver tissues from the NAFLD mouse model. Inhibition of miR-103-3p significantly alleviated the accumulation of lipid droplets in free fatty acid-treated L02 cells and liver tissues from mice with NAFLD. Inhibition of miR-103-3p reduced the contents of H_2_O_2_, TG, ALT, and AST and ROS production while increasing the ATP content. Moreover, the miR-103-3p antagomir alleviated liver tissue lesions in mice with NAFLD. Further studies identified ACOX1, a key enzyme for the oxidation and decomposition of fatty acids, as a direct target of miR-103-3p.

**Conclusions:**

These findings identified a negative regulatory mechanism between ACOX1 and miR-103-3p that promotes the pathogenesis of NAFLD and suggested that inhibition of miR-103-3p may be a potential treatment strategy for NAFLD.

## Introduction

Nonalcoholic fatty liver disease (NAFLD) develops into progressive nonalcoholic steatohepatitis (NASH), liver fibrosis, and cirrhosis and eventually causes liver cancer, which results in an increasing global public health burden that affects both adults and children [1, 2]. Studies on the pathogenesis of NAFLD in recent decades have revealed that NAFLD is initiated by aberrant lipid metabolism in the liver [3]. Aberrant lipid metabolism of the liver is caused by mitochondrial dysfunction, endoplasmic reticulum stress (ERS), oxidative stress, and activation of inflammation [4]. In addition to the classic factors, epigenetic mechanisms such as noncoding RNA are involved in the progression of NAFLD [5, 6]. Therefore, studying the mechanism of noncoding RNA in NAFLD may provide a promising strategy for NAFLD.

Noncoding RNA refers to RNAs such as rRNA, tRNA, snRNA, snoRNA, lncRNA, circRNA and miRNA that do not encode protein [7]. To date, 28,645 miRNAs that regulate one-third of human genes have been found in animals, plants and viruses [8]. MicroRNA is a type of 21–23 nt single-stranded small RNA that targets the 3′UTR of mRNAs and silences or degrades mRNA, which regulates the progression of NAFLD [9, 10]. Patients with NAFLD displayed dramatic upregulation of miR-132 expression, and suppression of miR-132 expression decreased lipid accumulation to improve NAFLD in vivo [11]. Intriguingly, miR-26a was decreased in NAFLD patients’ livers, and overexpression of miR-26a inhibited high-fat diet (HFD)-induced oxidative stress, lipid accumulation, activation of inflammation and hepatic damage [12]. Anti-miR-214-3p increased ULK1-induced autophagic activity to mitigate hepatic steatosis [13].

MiR-103-3p is an important member of the miRNA family and is related to the regulation of pathological processes such as osteoporosis, diabetes, and obesity [14, 15]. Recent research has shown that miR-103-3p is a noninvasive prospective biomarker for NAFLD diagnosis [16]. The increase in miR-103-3p is related to liver steatosis, a type of NAFLD [17]. However, the regulatory mechanism of miR-103-3p in NAFLD has not yet been studied.

In this research, we used HFD-fed mice and FFA-treated L02 cells to research the possible regulatory effect of miR-103-3p on NAFLD. MiR-103-3p expression in hepatic and hepatocellular tissues increased. Moreover, suppression of miR-103-3p alleviated NAFLD damage both in vivo and in vitro by regulating hepatic lipid metabolism. Finally, we found that acyl-CoA oxidase 1 (ACOX1) was the target gene of miR-103-3p.

## Materials and methods

### Cell culture and cellular steatosis model construction

Human normal liver L02 cells were purchased from ATCC and cultured in RPMI 1640 medium (Gibco™, A4192301) with 10% FBS (Gibco™, 12483020) and 1% penicillin/streptomycin in 5% CO_2_ in a 37 °C humidified atmosphere. We used 1 mM FFA [FFA; oleate acid and palmitate acid (2:1)] to induce L02 cells to establish a NAFLD cell model. L02 cells were cultured in RPMI 1640 medium with FBS until the cells reached 60–80% confluence and then treated with 1 mM FFA for 48 hours (48 h).

### MiRNA and small interfering RNA transfection

L02 cells were treated with FFA for 24 h, and then, Antagomir-103-3p (300 µM) transfection was performed, followed by incubation with FFA for 24 h. Antagomir-103-3p (5′-UCAUAGCCC UGUACAAUGCUGCU-3′, GenePharma) and Agomir-103-3p (5′-AGCCGCCUUGUACAGGGCUAUGA-3′ GenePharma) were transfected via Lipofectamine 2000 (Invitrogen, 11668027) according to the manufacturer’s protocol. Nonsense single-stranded RNA (5′-CAGUACUUUUGUGUAGUACAA-3′, GenePharma) was transfected into L02 cells as the negative control (NC) group.

### Animals and treatment

Six-week-old male C57BL/6 mice (Changsheng Biotechnology) were fed for 1 week and then randomly divided into four groups: (1) the control group; (2) the HFD group; (3) the HFD + 15 mg/kg Antagomir-NC; and (4) the HFD + 15 mg/kg Antagomir-103-3p. All animal studies were approved by the Animal Care and Use Committee of Zhejiang University in accordance with the Chinese guidelines for the care and use of laboratory animals.

### Western blot assay

A lysis buffer (Thermo Scientific™, PV5598) containing PMSF (Thermo Scientific™, 36978) was used to lyse L02 cells. Then, the BCA method (Thermo Scientific™, 5000006) was used to determine the protein concentration. After 40 µg of protein sample was mixed with 5× sample buffer, the proteins were separated by SDS-PAGE and transferred to PVDF membranes (Bio-Rad, 162-0177). After the membranes were blocked with 4% milk for 2 h, the following antibodies were added and incubated overnight at 4 °C: FASN antibody (1:500, Proteintech, 10624-2-AP), ACSL1 antibody (1:1000, Affinity, DF9605), ACOX1 antibody (1:1000, Affinity, DF12046), and GAPDH antibody (1:2500, Abcam, AB9485). After the membrane was washed 3 times with PBS, HRP secondary antibody was added and incubated for 2 h at room temperature. ECL (Bio-Rad, 170-5060) was added to the membrane, which was placed in the GelDoc imaging system (Bio-Rad). ImageJ software was used to perform optical density analysis.

### Dual-luciferase reporter assay

According to the predicted miR-103-3p binding sites of ACOX1, wild-type ACOX1 (ACOX1-WT) and mutant-type ACOX1 (ACOX1-MT) were synthesized (GenePharma) and inserted into the luciferase reporter pGL3-control vector. Luciferase reporter plasmids ACOX1-WT or ACOX1-MT were transfected into 293T cells with Agomir-103-3p or Agomir-NC for 48 h. Then, the luciferase activity was measured by a Glomax 20/20 luminometer.

### qRT-PCR assays

Total RNA was extracted by TRIzol reagent (Invitrogen™, A33251), and a TaqMan™ MicroRNA Reverse Transcription Kit (Applied Biosystems™, 4366596) or First Strand cDNA Synthesis Kit (Thermo Scientific™, K1632) was used to reverse transcribe complementary DNA (cDNA) for miRNAs or mRNA, respectively. qRT–PCR experiments of *miR-103-3p*, *FASN*, *ACOX1*, and *ACSL1* were performed using the StepOnePlus Real-Time PCR system with Maxima SYBR Green qPCR Master Mixes. The relative expression of genes was calculated by the 2^−△△Ct^ method. The qRT-PCR primer sequences were shown in Table 1.

**Table 1:** The qRT-PCR primer sequences

Gene	Forward primer (5′–3′)	Reverse primer (5′–3′)
Mus-FASN	GGTTACACTGTGCTAGGTGTTG	TCCAGGCGCATGAGGCTCAGC
Mus-ACOX1	CCTGATTCAGCAAGGTACGG	TCGCAGACCCTGAAGAAATC
Mus-ACSL1	TGGGGTGGAAATCATCAGCC	CACAGCATTACACACTGTACAACGG
Human-ACSL1	AACAGACGGAAGCCCAAGC	TCGGTGAGTGACCATTGCTC
Human-FASN	AACTCCAAGGACACAGTCACCAT	CAGCTGCTCCACGAACTCAA
Human-ACOX1	TGTCCTATTTGAACGACCTGCCCA	AGGTTCCAAGCTACCTCCTTGCTT
Mus-GAPDH	TGCACCACCAACTGCTTAG	GGATGCAGGGATGATGTTC
Human-GAPDH	GCTGGCGCTGAGTACGTCGTGGAGT	CACAGTCTTCTGGGTGGCAGTGATGG
Human-miR-103-3p	ACACTCCAGCTGGGAGCAGCATTGTAC	TGGTGTCGTGGAGTCG
Mus-miR-103-3p	ACACTCCAGCTGGGAGCAGCATTGTAC	TGGTGTCGTGGAGTCG
Mus-U6	CTCGCTTCGGCAGCACA	AACGCTTCACGAATTTGCGT
Human-U6	GCGCGTCGTGAAGCGTTC	GTGCAGGGTCCG AGGT

#### Oil red O staining

Then, 10% paraformaldehyde was applied to fix the slides, and Oil red O staining solution (Sigma, SLBP5248V) was added to the slides for 15 min. After washing with 60% isopropanol, the slides were counterstained with haematoxylin and imaged by light microscopy.

#### Biochemical analysis and measurement of ROS

The triglyceride (TG), alanine aminotransferase (ALT), aspartate transaminase (AST), adenosine triphosphate (ATP) and hydrogen peroxide (H_2_O_2_) levels in vitro and in vivo were measured using a triglyceride reagent (Nanjing Jiancheng Bioengineering Institute (NJBI), A110-1), ALT Activity Assay (NJBI, C009-2), AST Activity Assay Kit (NJBI, C101-2), ATP test kit (NJBI, A095) and Hydrogen Peroxide Assay (NJBI, A064-1) according to the manufacturer’s instructions. Staining with DCF-DA was performed using a Total ROS Assay Kit (Invitrogen™, 88-5930-74), and ROS production in the cells and mouse liver tissues was analysed by a BD FACSCanto II cytometer (BD Biosciences).

#### Haematoxylin and eosin (HE) staining

Liver tissues were fixed with 4% paraformaldehyde, and 5-µm-thick tissue sections were stained with HE. After air-drying, the slides were mounted with neutral gum, and the morphological changes in the liver tissues were detected under a light microscope.

#### Statistical analysis

Data were analysed by SPSS 18.0 and are presented as the mean ± standard deviation (SD). The means between groups were evaluated using Student’s t test or one-way ANOVA. P values < 0.05 were considered significant.

## Results

### MiR-103-3p expression increased in NAFLD, and suppression of miR-103-3p improved the NAFLD cell phenotype

**Fig. 1:**
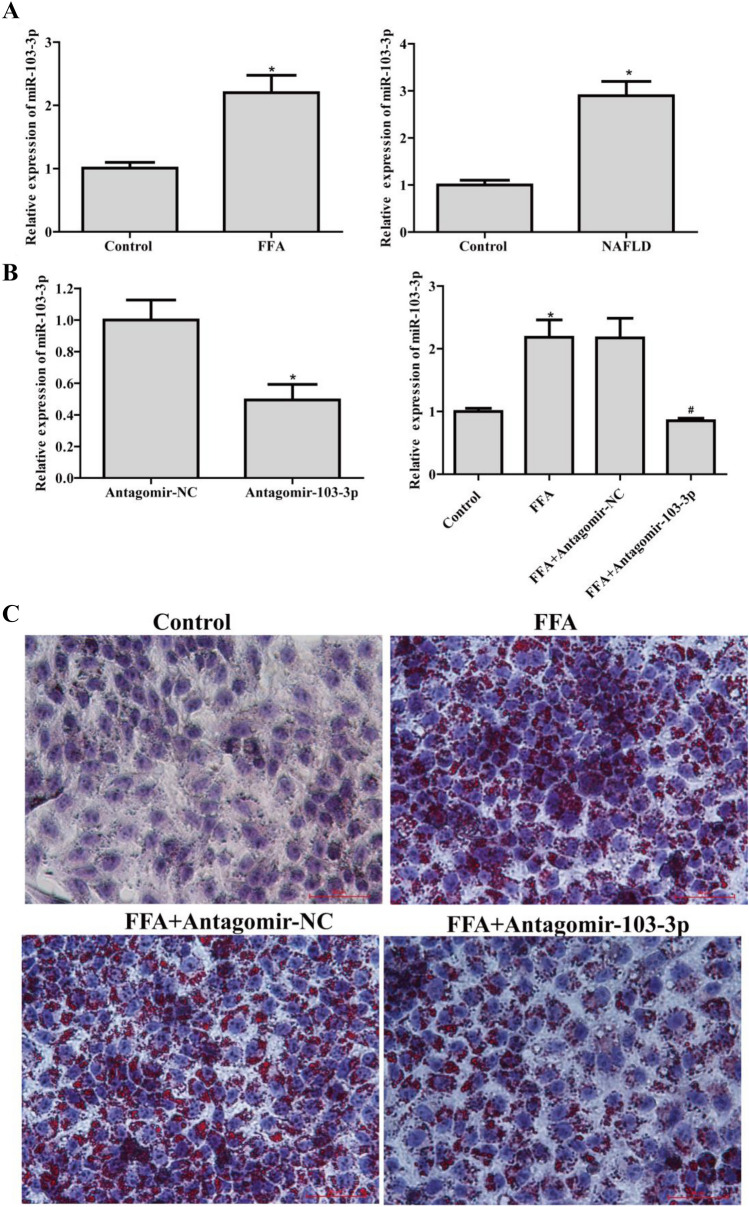
MiR-103-3p expression increased in NAFLD, and Antagomir-103-3p improved the NAFLD cell phenotype. **A** qRT-PCR detected miR-103-3p expression in FFA-treated L02 cells and liver tissues from the mice with NAFLD. **B** FFA was used to treat L02 cells for 24 h, followed by Antagomir-NC or Antagomir-103-3p transfection and incubation with 1 mM FFA for 24 h; qRT-PCR assays detected the expression of miR-103-3p. **C** Oil Red O staining assays detected lipid droplet accumulation in L02 cells. *P < 0.05 compared with the control group; #P < 0.05 compared with the FFA+Antagomir-NC group

qRT-PCR assays showed that miR-103-3p expression was increased in the FFA-treated cells and liver tissues from mice with NAFLD (Fig. 1A). These results showed that miR-103-3p may regulate the development of NAFLD. To determine the role of miR-103-3p in the pathological process of NAFLD, we successfully constructed an Antagomir-103-3p that downregulated miR-103-3p expression in the FFA-treated L02 cells (Fig. 1B). Oil Red O staining assays showed that Antagomir-103-3p significantly alleviated the accumulation of lipid droplets in NAFLD group cells (Fig. 1C).

### Suppression of miR-103-3p alleviated the damage to NAFLD group cells

**Fig. 2:**
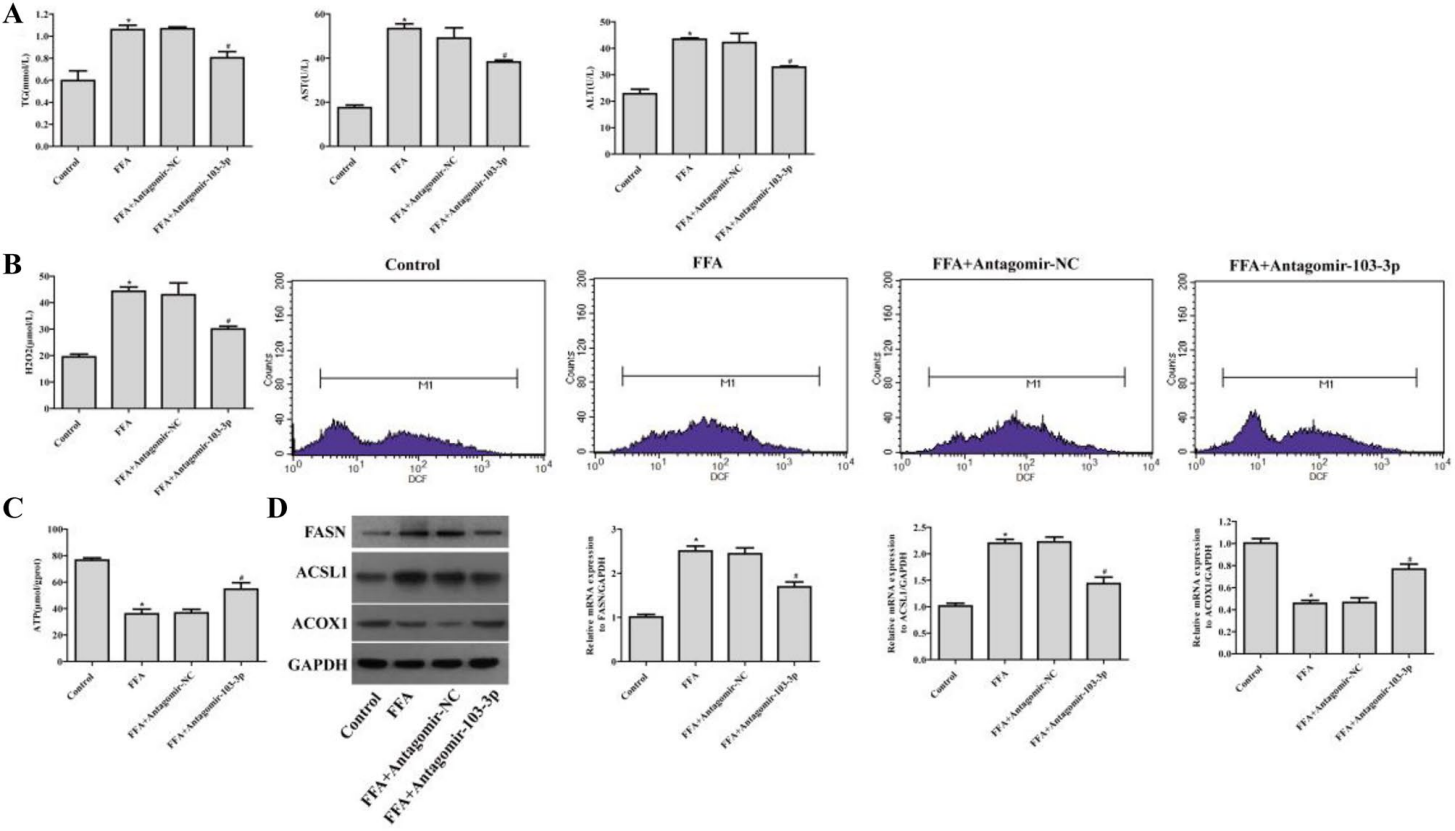
Antagomir-103-3p alleviated the abnormal lipid metabolism and inflammation of cells in the NAFLD group. **A** The TG, ALT and AST contents in L02 cell supernatant were examined by biochemical tests. **B** The content of H_2_O_2_ and ROS generation were examined in L02 cell supernatants and L02 cells, respectively. **C** The ATP content in L02 cells was examined by biochemical tests. **D** The protein and mRNA levels of ACOX1, FASN and ACSL1 were examined by western blotting and qRT-PCR, respectively. *P < 0.05 compared with the control group; ^#^P < 0.05 compared with the FFA+Antagomir-NC group

Abnormal lipid metabolism results in hepatic steatosis in NAFLD [18]. The biochemical test results showed that compared with the Antagomir-NC-treated cells, the Antagomir-103-3p-treated NAFLD group cells had significantly decreased TG, ALT, and AST contents (Fig. 2A). Moreover, the suppression of miR-103-3p decreased the H_2_O_2_ content and ROS production (Fig. 2B). The ATP content was significantly increased in the Antagomir-103-3p-treated NAFLD group cells (Fig. 2C). In addition, qRT-PCR and western blot results showed that NAFLD model cells treated with Antagomir-103-3p displayed inhibition of fatty acid synthase (FASN) and long-chain acyl coenzyme A synthase 1 (ACSL1) mRNA levels and FASN and ACSL1 protein expression while promoting ACOX1 mRNA and protein expression (Fig. 2D). The above findings revealed that suppression of miR-103-3p significantly alleviates inflammation, abnormal lipid metabolism, oxidative stress and damage to cells in the NAFLD group.

### Suppression of miR-103-3p alleviated the damage to mice with NAFLD

**Fig. 3:**
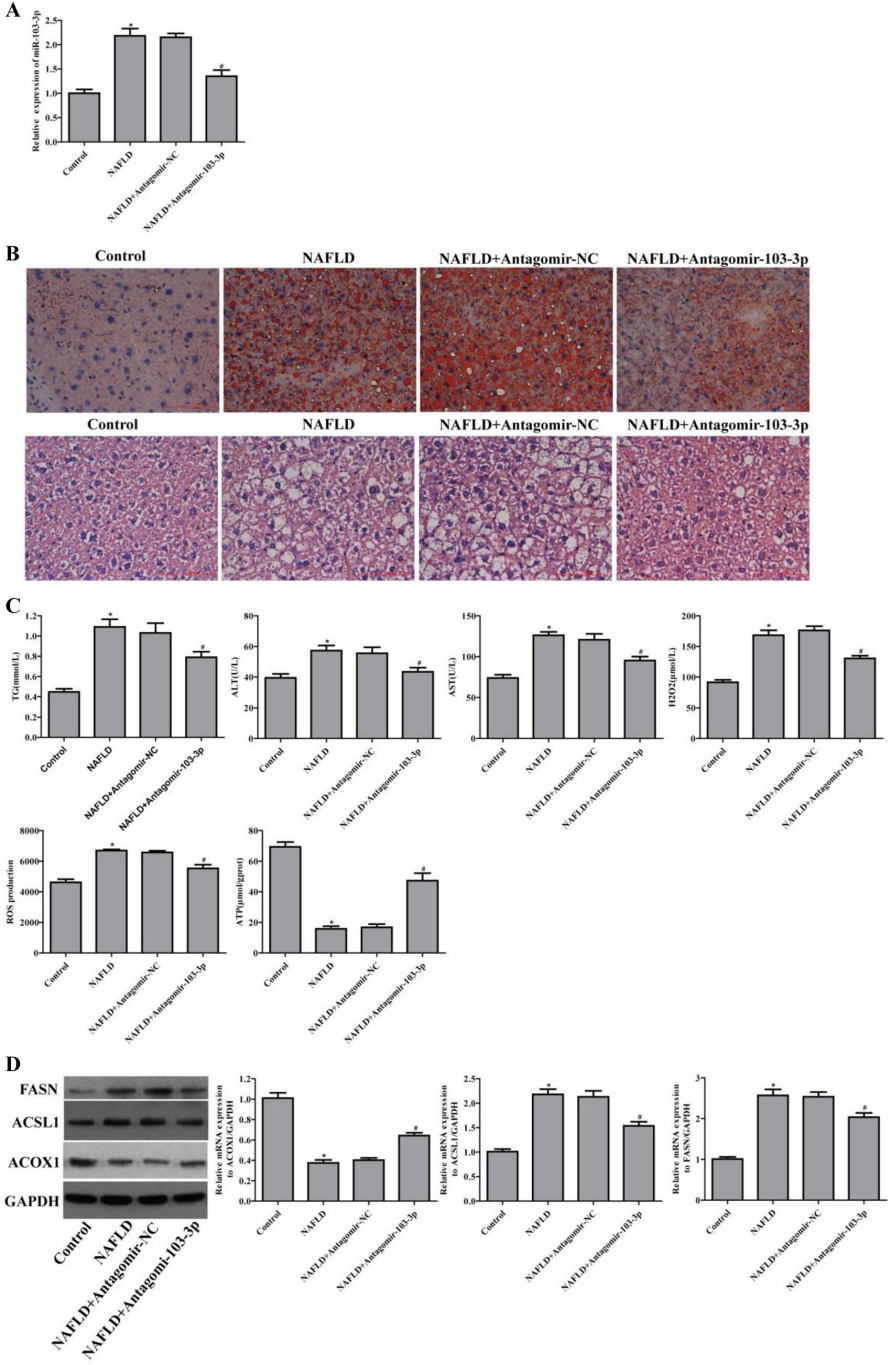
Antagomir-103-3p alleviated the damage to mice with NAFLD. Mice with NAFLD were fed an HFD for 8 weeks, and Antagomir-NC or Antagomir-103-3p was used for tail vein injection once a week for 2 weeks. **A** MiR-103-3p expression in mouse liver tissues was examined by qRT-PCR. **B** Oil Red O staining detected lipid droplet accumulation in mouse liver tissues, and HE staining detected liver tissue lesions in mice. **C** The TG, ALT, AST and H_2_O_2_ contents in mouse serum were examined, while ROS generation and ATP content were examined in mouse tissues. **D** The protein and mRNA levels of ACOX1, FASN and ACSL1 were examined by western blotting and qRT-PCR, respectively. *P < 0.05 compared with the control group; ^#^P < 0.05 compared with the NAFLD+Antagomir-NC group

To research the correlation between miR-103-3p and NAFLD in vivo, we constructed a mouse model of NAFLD with an HFD for 8 weeks, and Antagomir-103-3p was used for tail vein injection to interfere with miR-103-3p expression. The qRT-PCR results showed that Antagomir-103-3p effectively downregulated miR-103-3p expression in liver tissue from the mice with NAFLD (Fig. 3A). Oil Red O staining revealed that the number of red-stained lipid droplets increased in the liver tissue of the mice with NAFLD. The number of red-stained lipid droplets in the liver tissue of the mice with NAFLD in the Antagomir-103-3p group was reduced. HE staining revealed that the liver tissues of the mice with NAFLD had increased steatosis hepatocytes, ballooning degenerated hepatocytes and visible focal necrosis, and Antagomir-103-3p alleviated liver tissue lesions in the mice with NAFLD (Fig. 3B). In summary, Antagomir-103-3p improves the pathological changes in fatty liver tissue, indicating that miR-103-3p is related to NAFLD development.

The biochemical test results showed that the serum TG, ALT, and AST contents of the Antagomir-103-3p-treated mice with NAFLD were significantly reduced. Moreover, Antagomir-103-3p inhibited ROS production and H_2_O_2_ in the liver tissues and serum of the mice with NAFLD. The ATP content was significantly increased in the liver tissues of the Antagomir-103-3p-treated mice with NAFLD (Fig. 3C). In addition, Antagomir-103-3p inhibited FASN and ACSL1 mRNA levels and FASN and ACSL1 expression in liver tissues from the mice with NAFLD while promoting ACOX1 mRNA levels and ACOX1 expression (Fig. 3D). The above results revealed that suppression of miR-103-3p significantly alleviates inflammation, abnormal lipid metabolism, oxidative stress and damage in the mice with NAFLD.

### ACOX1 is the target gene of miR-103-3p

**Fig. 4:**
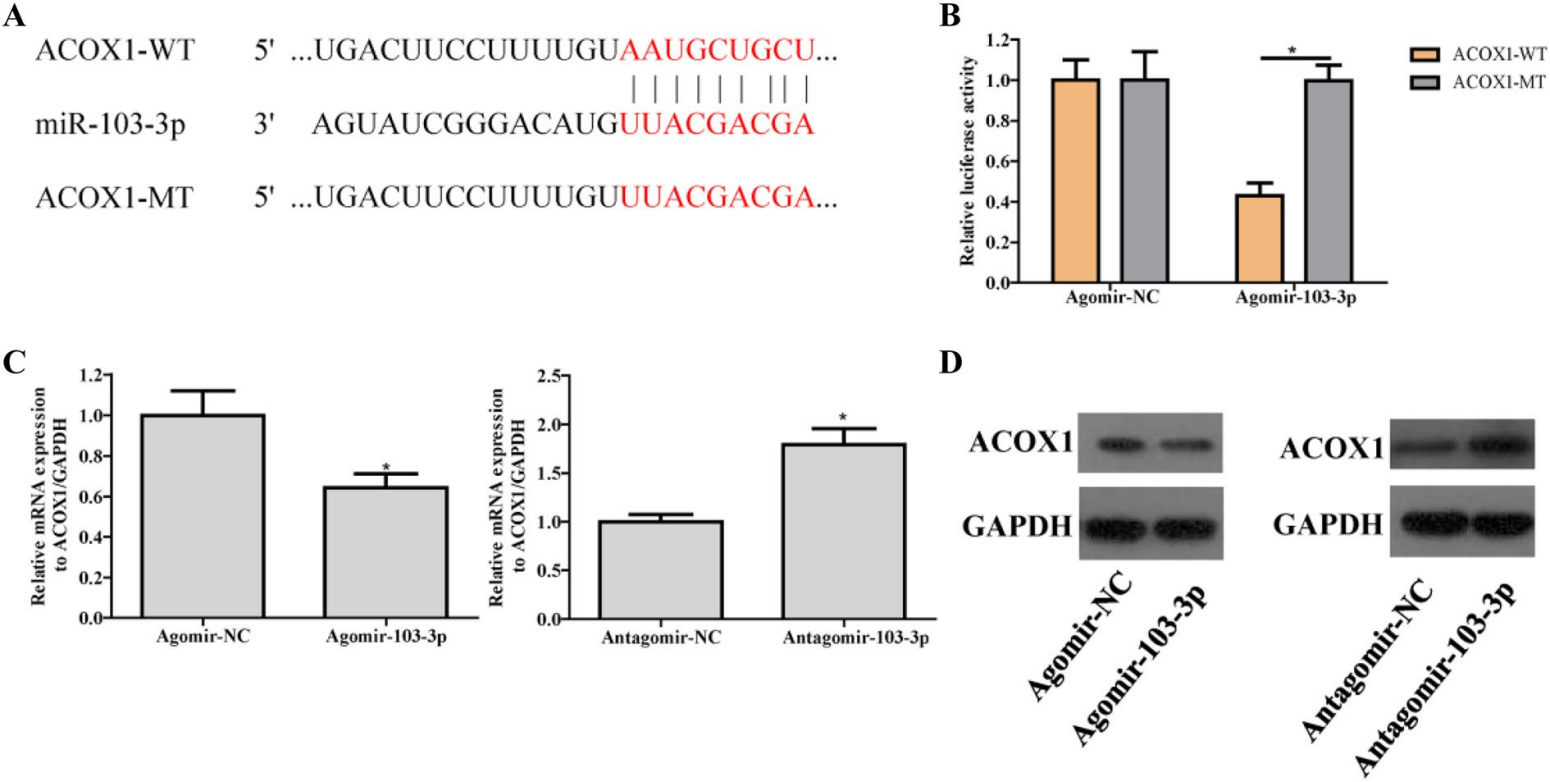
ACOX1 is a target gene of miR-103-3p. **A** The binding sites between miR-103-3p and ACOX1 were predicted via TargetScan. **B** The binding of miR-103-3p to the ACOX1 3′UTR was verified by a dual luciferase reporter assay. **C** The ACOX1 mRNA level was determined by qRT-PCR. **D** ACOX1 protein expression was determined by western blots. The data are expressed as the mean ± standard deviation, *P < 0.05

The TargetScan website was used to predict the potential target genes of miR-103-3p and find the binding sites between miR-103-3p and ACOX1 (Fig. 4A). Dual luciferase reporter assays showed that miR-103-3p targeted ACOX1 (Fig. 4B). Moreover, qRT-PCR showed that Agomir-103-3p downregulated ACOX1 mRNA expression, while Antagomir-103-3p upregulated ACOX1 mRNA expression (Fig. 4C). Western blots also showed that ACOX1 expression decreased in the Agomir-103-3p-treated cells, while ACOX1 expression increased in the Antagomir-103-3p-treated cells (Fig. 4D). The above results showed that miR-103-3p targeted ACOX1.

**Figure:**
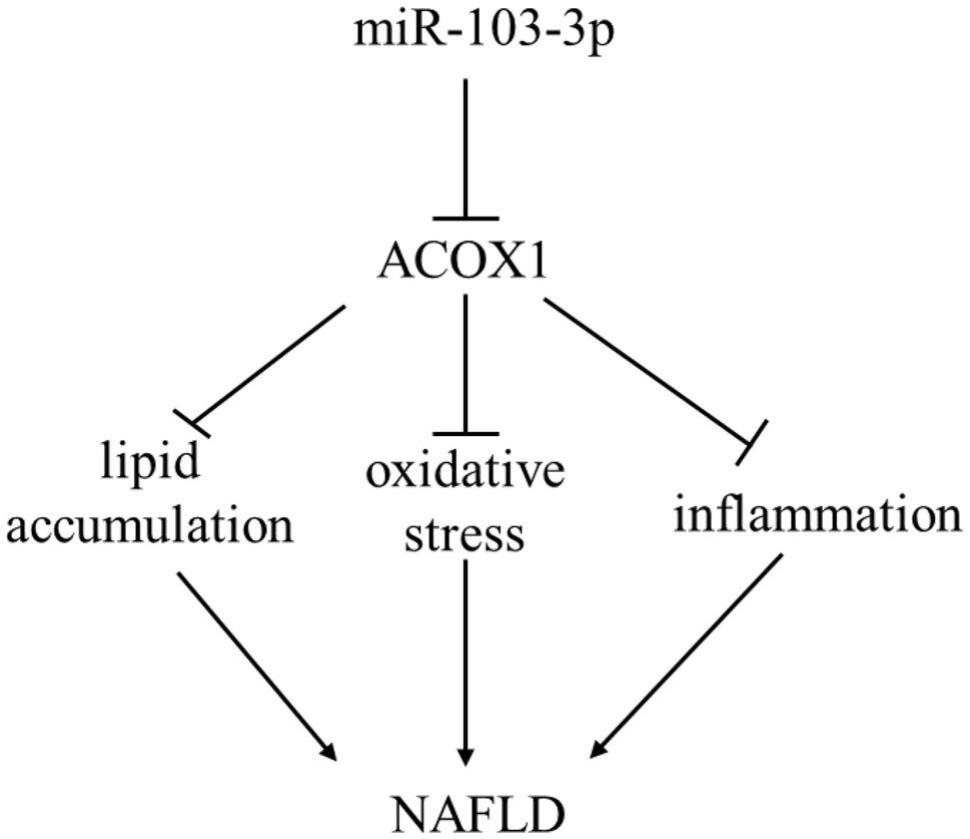
A model for miR-103-3p regulation of NAFLD by targeting ACOX1 (see text for details)

## Discussion

NAFLD is characterized by abnormal lipid accumulation in hepatocytes in the absence of alcohol intake [19]. In particular, NAFLD can progress to advanced NASH, fibrosis and cirrhosis and even hepatic carcinoma, which is a threat to human health [20]. However, there is no effective drug for NAFLD therapy. MiRNAs as a noninvasive marker may show promise in the diagnosis of NAFLD by replacing liver biopsy and provide a new strategy for NAFLD [21, 22]. The expression of miR-103-3p is related to steatosis activity, fibrosis score, stages, and prognostic markers of NAFLD [16]. We found that miR-103-3p was increased in the FFA-treated hepatocytes and liver tissues of the mice with NAFLD and may be a prospective biomarker for NAFLD diagnosis.

Abnormal lipid accumulation in hepatocytes results in TG deposition, upregulation of the levels of H_2_O_2_, ALT and AST and decreases in ATP [23–25]. Excessive TG accumulation is a feature of NAFLD, leading to hepatic lipid droplets [26]. We found that suppression of miR-103-3p alleviates lipid droplet accumulation in NAFLD group cells and liver tissues of the mice with NAFLD, accompanied by an increase in TG content. ALT and AST were increased in the NAFLD group compared to the control group [27]. We found that suppression of miR-103-3p downregulated ALT and AST levels in NAFLD cell supernatant and mouse serum. Mitochondrial oxidative function has a key role in the development of NAFLD, and fatty acid oxidation, ATP synthesis and ROS production influence lipogenesis and gluconeogenesis [28]. Consistent with our finding, ATP was significantly increased by suppression of miR-103-3p. Moreover, suppression of miR-103-3p decreased ROS production in NAFLD group cells and liver tissues from the mice with NAFLD. However, the specific mechanism by which miR-103-3p regulates NAFLD damage requires further study.

MiRNAs target the 3′UTR of mRNAs and silence or degrade mRNA, which regulates the progression of NAFLD [29]. MiR-17 targets Pknox1 to reduce hepatocyte steatosis by inhibiting intracellular TG and lipid accumulation [30]. MiR-130b-5p targets IGFBP2 to upregulate SCD1, ACC1 and FAS expression, and suppression of miR-130b-5p prevented lipid accumulation in hepatocytes [31]. Overexpression of miR-183-5p inhibited Btg1 to upregulate lipogenic gene expression [32]. We found that miR-103-3p regulates the mRNA levels and protein expression of the key enzymes for fatty acid synthesis (FASN and ACSL1) and the oxidation and decomposition of fatty acids (ACOX1) to alleviate liver tissue lesions in NAFLD. Moreover, miR-103-3p targets ACOX1 to modulate the development of NAFLD. ACOX1 regulates lipid homeostasis, oxidative stress, and hepatic inflammation, and the suppression of ACOX1 regulated the accumulation of TG in NAFLD [33, 34]. The miR-31-5p-ACOX1 axis was shown to alter lipid metabolomes in oral squamous cell carcinoma [35]. MiR-103a-3p targets HMGB1 to alleviate LPS-induced inflammation [36]. Moreover, ACOX1 was verified to be the specific target of miR-103-3p by qRT-PCR, western blot and dual-luciferase assays. Our research showed that miR-103-3p may inhibit ALT and AST to improve inflammation, decrease ROS and H_2_O_2_ levels to improve oxidative stress, and increase ATP levels to alleviate NAFLD damage by targeting ACOX1.

## Conclusions

In summary, we demonstrated the possible regulation of miR-103-3p in NAFLD. MiR-103-3p expression was increased in the FFA-treated cells and liver tissues from the mice with NAFLD. Suppression of miR-103-3p alleviates abnormal lipid metabolism, oxidative stress and NAFLD damage by targeting ACOX1. Our research may provide a prospective biomarker for NAFLD diagnosis and a new strategy for NAFLD.

## Data Availability

The data of the study are available from the corresponding author upon reasonable request.
